# Viral tropism in plants, reproductive tissues, and seeds

**DOI:** 10.1007/s00203-025-04353-9

**Published:** 2025-05-23

**Authors:** Leandro Alberto Núñez-Muñoz, Berenice Calderón-Pérez, Roberto Ruiz-Medrano, Beatriz Xoconostle-Cázares, Rodolfo de la Torre-Almaraz

**Affiliations:** 1 Facultad de Estudios Superiores Iztacala, Universidad Nacional Autónoma de México, Unidad de Biotecnología y Prototipos, Avenida de los Barrios #1, Los Reyes Iztacala, 54090 Tlalnepantla, State of Mexico Mexico; 2 Circuito de los Posgrados S/N, Universidad Nacional Autónoma de México, Posgrado en Ciencias Biológicas, Ciudad Universitaria, Coyoacán, 04510 Mexico City, Mexico; 3 Centro de Investigación y de Estudios Avanzados del Instituto Politécnico Nacional (CINVESTAV), Departamento de Biotecnología y Bioingeniería, Av. Instituto Politécnico Nacional 2508, 07360 Mexico City, Mexico; 4 Centro de Investigación y de Estudios Avanzados del Instituto Politécnico Nacional (CINVESTAV), Programa de Doctorado Transdisciplinario en Desarrollo Científico y Tecnológico Para la Sociedad, Av. Instituto Politécnico Nacional 2508, 07360 Mexico City, Mexico

**Keywords:** Plant virus, Seed transmission, Viral accumulation, Viral movement, Viral tropism

## Abstract

Plant viral tropism refers to virus ability for infecting and replicating within specific cell types, tissues or hosts. Plant viral tropism is shaped by the absence of specific membrane-associated viral receptors and the supracellular nature of viral transport through plasmodesmata and vascular tissues. This review focuses on the molecular and cellular determinants of plant viral tropism, including modifications in plasmodesmal permeability, host-mediated RNA silencing, and tissue-specific viral protein localization. We discuss how certain viruses target reproductive organs, meristems, and seeds, overcoming antiviral barriers to establish persistent infections. Additionally, we explore the role of host factors in shaping viral distribution. Advances in super-resolution microscopy, single-cell transcriptomics, and proteomics have significantly expanded our ability to dissect virus-host interactions at the nanoscale, uncovering new mechanisms of viral accumulation. Understanding these processes is essential not only for improving crop resistance and designing integrated disease management strategies, but also for repurposing plant viruses as tools for targeted delivery and biotechnological applications.

**Supplementary Information:**

The online version contains supplementary material available at 10.1007/s00203-025-04353-9.

## Introduction

Viruses are among the most economically significant plant pathogens, causing annual agricultural losses exceeding $30 billion (Jones and Naidu 2019). Their impact on crop production, which is essential for human and animal nutrition, poses a threat to global food security (Rybicki 2015). The field of virology emerged from the pioneering work of Dmitri Ivanovsky and Martinus Beijerinck in the late nineteenth century, who demonstrated the transmission of tobacco disease through finely filtered extracts of infected plants, leading to the discovery of Tobacco mosaic virus (TMV, *Tobamovirus tabaci*) (Bos 1999). Since then, advances in plant virology have significantly enhanced our understanding of viral replication and transmission mechanisms. Nevertheless, fundamental aspects of plant virus biology, particularly the mechanisms underlying tropism patterns and differential accumulation in reproductive and seed tissues, remain underexplored. Plant viruses exploit supracellular transport networks for their movement, spreading between cells through plasmodesmata and systemically via the phloem. However, this mode of transport poses significant challenges in identifying tissue-specific sites of viral accumulation. This review synthesizes the current knowledge on plant viral tropism, focusing on virus entry, accumulation, and systemic movement through the phloem. Elucidating the mechanisms underlying viral localization and seed transmission in plants has significant implications for agricultural practices and disease management.

## The role of viral tropism in entry and movement

**Fig. 1:**
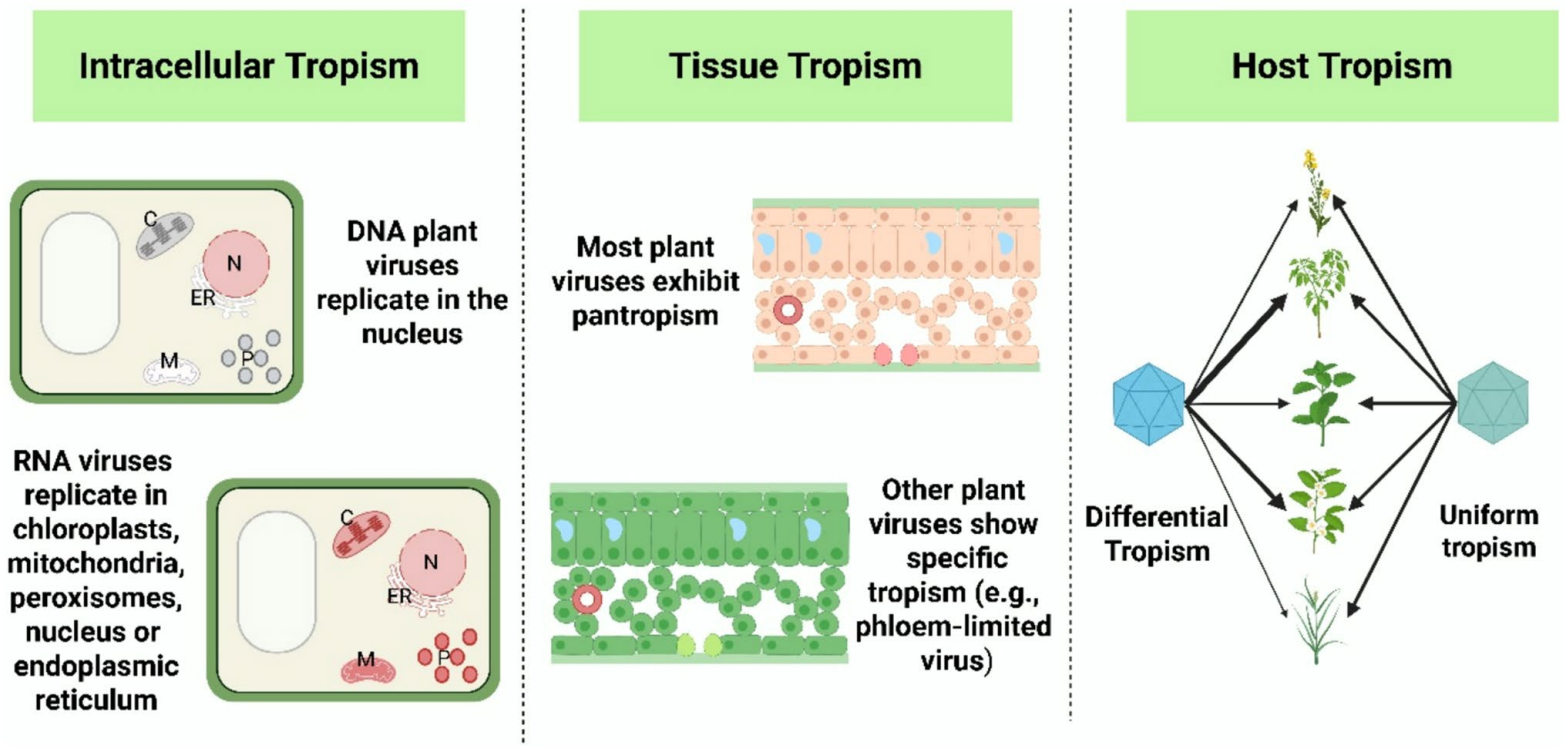
Classification of Plant Viral Tropism. Tropism in plant viruses can be categorized into intracellular tropism, tissue tropism, and host tropism. Intracellularly, DNA plant viruses replicate in the nucleus. RNA viruses replicate in chloroplasts (C), mitochondria (M), peroxisomes (P), nucleus (N) or endoplasmic reticulum (ER). At the tissue level, most plant viruses exhibit pantropism, infecting multiple tissue types, although some are restricted to specific tissues (e.g. phloem-limited viruses). Host tropism refers to the virus differential ability to infect and replicate in distinct plant hosts. Some viruses exhibit differential host tropism, accumulating to different levels across hosts within their range, while others show uniform tropism with comparable infection efficiency across hosts. In the image, infected organelles and tissues are highlighted in pink to enhance visual clarity

Plant viral tropism refers to the ability of a virus to preferentially infect and replicate within specific cell types or organelles, tissues or hosts while encountering restrictions in others (McFadden et al. 2009; Singhal et al. 2021) (Fig. 1). In plants, viral entry must overcome the cell wall, which acts as a physical barrier restricting the diffusion of macromolecules larger than 60 kDa and limiting direct access to the plasma membrane (Buchmann and Holmes 2015). Plant viruses bypass this barrier by exploiting mechanical damage or employing vector organisms to introduce viral particles directly into plant tissues.

**Fig. 2:**
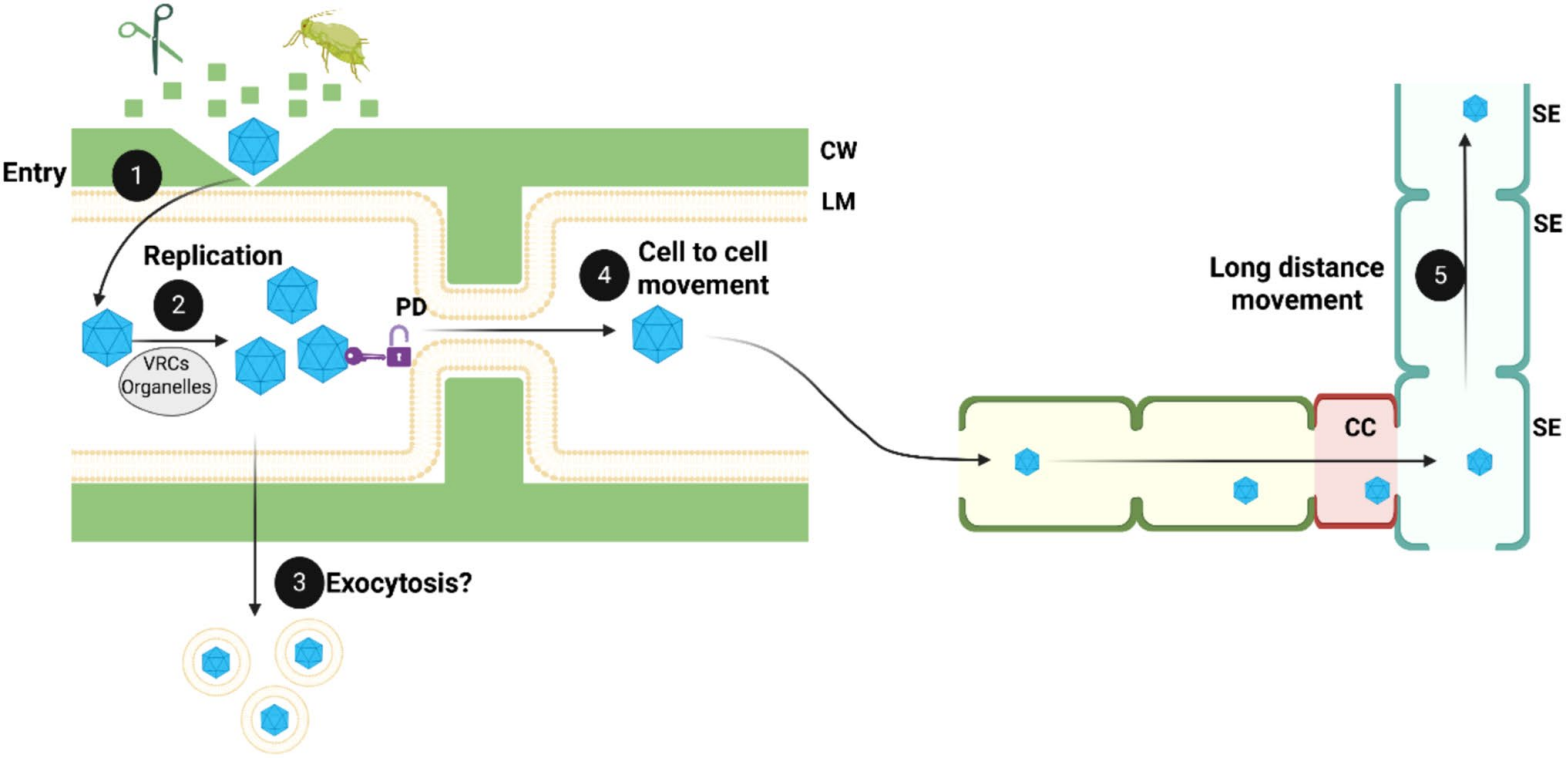
Generalized model of plant viral infection. (1) Viral entry into host cells occurs through mechanical damage or vector-mediated transmission, bypassing the cell wall (CW) and lipid membrane (LM). (2) Upon entry, replication occurs in viral replication complexes (VRCs) or host organelles. (3) Some viral particles or ribonucleoprotein complexes have been detected in the apoplast, suggesting possible extracellular release through exocytosis or secretion via extracellular vesicles. (4) Plant viruses move between adjacent cells through plasmodesmata (PD), often requiring viral movement proteins to increase size exclusion limits. (5) Long-distance movement is achieved via the phloem, with plant viruses entering companion cells (CC) and subsequently loading into sieve elements (SE) for systemic transport

In contrast to animal viruses, plant viruses do not rely on plasma membrane receptors for cell entry, due to the presence of the cell wall. Instead, entry typically occurs via mechanical damage or is facilitated by vectors that bypass the cell wall barrier (Fig. 2; step 1). Receptor-mediated endocytosis is not a primary entry route in plants. However, some host proteins involved in vesicle trafficking, such as dynamin-related proteins and synaptotagmins, have been linked to viral replication and intracellular movement. Whether viruses actively exploit endocytosis remains uncertain (Uchiyama et al. 2014; Wu et al. 2020).

Plant virus replication is a coordinated process that depends on the formation of membrane-associated viral replication complexes (VRCs), which vary in localization depending on the viral species (Fig. 2; step 2). Positive-strand RNA viruses, the largest group of plant viruses, typically replicate on modified membranes derived from organelles such as the endoplasmic reticulum, peroxisomes, chloroplasts, or mitochondria (Incarbone et al. 2021). Most negative-strand RNA viruses, such as Bunyavirales and Mononegavirales, also replicate within membrane-bound VRCs, utilizing similar strategies involving protein–membrane and protein–RNA interactions (German et al. 2020). However, some negative-strand RNA viruses in plants, such as nucleorhabdoviruses, replicate in the nucleus (Dietzgen et al. 2017). In contrast, DNA viruses like geminiviruses replicate within the host nucleus, hijacking the cellular DNA replication machinery (Arroyo-Mateos et al. 2018).

Recent studies have detected viral particles within the apoplast, suggesting that exosomal trafficking may contribute to virus release into the extracellular space (Movahed et al. 2019; Hu et al. 2021) (Fig. 2, step 3). In that sense, mechanistic insights into extracellular vesicles (EVs) in plant–virus interactions remain limited, but recent evidence suggests a dual functional role. For example, Turnip mosaic virus (TuMV, *Potyvirus rapae*) replication complexes—including viral RNA and proteins—have been detected within EVs isolated from *Nicotiana benthamiana* leaves (Movahed et al. 2019). Proteomic analysis revealed the presence of viral replication factors, infection-related proteins and immune signaling molecules, suggesting that EVs may facilitate viral spread or act as carriers of pathogen-associated molecular patterns that trigger systemic immune responses. Although the functional characterization of EVs in plants remains at an early stage, these observations reveal shared features with animal systems and highlight the need for further investigation into their role in viral tropism and immune modulation (Zhou et al. 2022).

Interestingly, several families of enveloped plant viruses, such as *Rhabdoviridae*, *Tospoviridae*, and *Fimoviridae*, present a unique adaptation within their transmission cycle: they require interaction with lipid membranes in their insect vectors to complete successful infection and transmission (Stavolone and Lionetti 2017; Helenius 2018). This lipid-dependence resembles the endocytic pathways used by many animal viruses and suggests a molecular convergence in membrane-interaction strategies across kingdoms. These membrane-mediated processes contribute to the tissue and vector tropism of enveloped plant viruses and underscore their biological distinction from non-enveloped viruses.

In this context, certain cuticular proteins such as Stylin-01 and MPCP4, located in the aphid stylet, have been identified as attachment factors used by Cauliflower mosaic virus (CaMV, *Caulimovirus tessellobrassicae*) to mediate virion retention or adhesion to the vector surface, without directly participating in intracellular entry. In contrast, several membrane-bound cellular receptors have been characterized on insect vectors that facilitate viral internalization and systemic dissemination. For example, alanyl aminopeptidase N in whiteflies acts as a receptor for Tomato yellow leaf curl virus (*Begomovirus coheni*), promoting endocytic uptake. Similarly, sugar transporter 6 and HSC70 proteins in leafhoppers serve as receptors for Rice stripe virus (*Tenuivirus oryzaclavatae*) and Rice gall dwarf virus (*Phytoreovirus betaoryzae*), respectively, enabling viral translocation across midgut and salivary gland barriers (Jangra et al. 2024).

Once inside the host, plant viruses move intercellularly through the symplasm by forming ribonucleoprotein complexes or tubules that modify the size exclusion limit (SEL) of plasmodesmata (PD) (Schoelz et al. 2011; Heinlein 2015) (Fig. 2; step 4). Many plant viruses, including *Tobamovirus* and *Cucumovirus,* also induce the expression of host enzymes such as β-glucanases and pectin methylesterases, which dilate the SEL of the PD to enhance viral transport (Chen et al. 2000; Huang et al. 2020). Structurally, PDs consist of a plasma membrane-lined pore, a desmotubule derived from the endoplasmic reticulum, and a cytoplasmic sleeve that determines the SEL. PDs vary in complexity, with primary PDs forming during cytokinesis and secondary PDs arising de novo. Their architecture (simple, branched or complex) influences viral spread, as meristematic tissues contain narrow SELs that restrict viral movement, while differentiated and vascular tissues have branched PDs that facilitate cell-to-cell movement (Bayer and Benitez-Alfonso 2024). Additionally, structural regulators such as multiple C2-domain transmembrane proteins and synaptotagmins can act as PD stabilizers and may facilitate viral trafficking (Levy et al. 2015; Brault et al. 2019; Ishikawa et al. 2020).

Movement proteins (MPs) and other viral components, such as coat proteins (CPs), replication-related proteins, counter-defense proteins (i.e., viral silencing suppressors), and non-coding regions, are involved in intercellular movement depending on the viral species (Benitez-Alfonso et al. 2010). Some persistent viruses, such as *Totiviridae* and *Partitiviridae*, lack classical MPs but replicate in meristematic cells, bypassing conventional PD-mediated transport (Bradamante et al. 2021). When viruses reach phloem sheaths, companion cells and sieve elements serve as conduits enabling systemic viral movement into uninfected tissues (Lucas 2006) (Fig. 2; step 5).

Viral tropism is associated with the selective and directional translocation of photoassimilates through the phloem, which transports RNAs and proteins, including signaling molecules (Maule and Palukaitis 1991). The mobility of these RNAs in the phloem is mediated by proteins, sequence-specific motifs and transfer RNA-like structures, as observed in the RNA virus genomes of *Tymovirus, Tobravirus, Furovirus, Pomovirus, Pecluvirus, Tobamovirus, Bromovirus, Cucumovirus,* and *Hordeivirus* (Kehr and Kragler 2018; Wang et al. 2021a; Wu et al. 2022). These viral genomes often display a leaf-to-apex tropism and encounter regulated phloem accessibility controlled by the synthesis and deposition of callose [β-(1,3)-D-glucan], which modulates viral spread (Wang et al. 2021b). In general, systemic virus movement correlates with photoassimilate transport and sink-to-source transition (Roberts et al. 2007).

Despite the differences in host range and pathogenicity between plant and animal viruses, in vitro evidence suggests that immune responses to plant viruses occur in invertebrates and vertebrates (Balique et al. 2015). Indeed, some plant viruses can replicate in yeast and fungi, suggesting that most eukaryotes share common cellular machinery in which viruses, particularly RNA viruses, exploit for replication (Panavas and Nagy 2003; Mascia et al. 2014). Additionally, some mycoviruses have been experimentally shown to replicate in plant cells, challenging the previously assumed strict host specificity of fungal viruses (Nerva et al. 2017). A notable exception to this host specificity is Providence virus (PrV, *Alphacarmotetravirus providencense*), a non-enveloped insect RNA virus, which has demonstrated the ability to establish productive infections in plants, invertebrates, and vertebrates (Jiwaji et al. 2019). However, there is currently little evidence of productive replication in vertebrate hosts, and plant viruses are not considered pathogenic to animals or humans.

Some plant viruses have been identified in animal and human microbiota, likely as a result of dietary intake. However, there is no evidence to suggest that these viruses exhibit cellular tropism or can establish active infection in non-plant hosts (Zhang et al. 2005). In contrast, increasing evidence points to meaningful interactions between plant viruses and fungal organisms. Viral sequences with high similarity to members of traditionally plant-associated families such as *Benyviridae, Ophioviridae*, and *Virgaviridae* have been identified in fungal metatranscriptomes (Marzano et al. 2016), and recent studies have demonstrated that plant viruses can infect and replicate in fungi. For instance, Cao et al. (2022) reported that nearly 50% of fungal strains isolated from symptomatic vegetable leaves carried plant viruses. Although virus persistence diminished over time in culture, these findings suggest that nonpersistent acquisition or transient colonization by plant viruses in fungi may be common under natural conditions. An additional striking example of functional cross-kingdom interaction was reported by Márquez et al. (2007) who demonstrated that a fungal virus (Curvularia thermal tolerance virus) is required for a symbiotic fungus to confer heat tolerance to its plant host, forming a three-way mutualism, illustrating how viruses can influence plant physiology through non-plant hosts.

## Viral-encoded factors and their influence on tropism

Viral tropism is influenced by diverse genome-encoded factors that interact with host cellular machinery. In plant viruses, tropism is mediated by distinct viral components that perform diverse functions. MPs play an important role in viral tropism through their interactions with PD and their intrinsic roles in pathogenicity (Kumar and Dasgupta 2021). Quantitative RT-PCR analyses have revealed differential accumulation of transcripts encoding MP and CP in the form of subgenomic or segmented RNA, suggesting a specific transcriptional regulation for each molecule. For instance, early accumulation of subgenomic RNAs encoding MPs has been observed in TMV, whereas those encoding CP are produced later (Knapp and Lewandowski 2001). CPs are essential for virion formation, viral spread, and systemic movement of single-stranded RNA (ssRNA) viruses, including members of the *Alphamovirus, Bromovirus, Comovirus, Cucumovirus, Closterovirus, Potexvirus,* and *Potyvirus* genera (Tilsner and Oparka 2012; Hipper et al. 2013; Zhou et al. 2019). Interestingly, some plant viruses, such as Groundnut rosette virus (*Umbravirus arachidis*), Tomato yellow leaf curl virus (*Begomovirus coheni*), and Tomato bushy stunt virus (*Tombusvirus lycopersici*) are capable of long-distance movement even without a functional CP (Padidam et al. 1995; Ryabov et al. 2001; Qu and Morris 2002; Manabayeva et al. 2013). However, the C-terminal domain of the readthrough protein (P5), an extended version of the CP, is involved in phloem tropism of Potato leafroll virus (PLRV, *Polerovirus PLRV*) (Peter et al. 2009), highlighting the variable role of CPs in viral tropism across different viruses. These variations reflect different viral evolutionary strategies developed to optimize their dissemination and transmission.

Additionally, untranslated regions (UTRs) at the 5’ and 3' ends of viral genomes, as well as viral non-coding RNAs, play pivotal roles in replication, accumulation, and systemic movement (Pallas and García 2011). For example, in Tomato golden mosaic virus (TGMV, *Begomovirus solanumaureimusivi*), three genetic elements, including a non-coding region of the viral genome and one of two different coding regions, are responsible for its mesophyll tissue tropism (Morra and Petty 2000).

In viroids, all elements controlling replication, cell-to-cell and systemic movement reside within their RNA genome, which further underscores the centrality of genome-encoded factors in determining tropism (Miller et al. 2016). Cellular tropism of viroids is dictated by their genome sequence, structure, and interactions with the host machinery. In *Pospiviroidae*, the conserved rod-like secondary structure and central conserved region facilitate nuclear transport and replication via a rolling-circle mechanism driven by host RNA polymerase II. Conversely, *Avsunviroidae* possess complex RNA structures with catalytic ribozyme activity, enabling chloroplast targeting and replication through plastidial RNA polymerases (Navarro et al. 2021).

## Tissue-specific tropism in plant viruses

During infection, plant viruses can colonize a wide range of host tissues (pantropism) or remain restricted to specific tissues (specific tropism). Most plant viruses exhibit pantropism, infecting diverse cell types and host tissues (Harper et al. 2014). However, some plant viruses exhibit a specific tropism, which is influenced by the different metabolic and physiological characteristics of organs or tissues. Notably, plant shoots and roots differ substantially in their anatomical structures, cell compositions, and gene expression patterns. Furthermore, they are subjected to contrasting environmental conditions, with shoots exposed to above-ground factors and roots exposed to below-ground factors. For instance, phloem-limited viruses rely on the photoassimilate transport pathway to spread systemically throughout the plant (Lewis et al. 2022) (Supplementary Table 1). Additionally, over 39 plant viruses, including positive-sense ssRNA viruses from *Alphaflexiviridae*, *Benyviridae*, *Bromoviridae*, *Closteroviridae*, *Potyviridae*, *Secoviridae*, *Solemoviridae*, *Tombusviridae,* and *Virgaviridae* families, have been reported to enter the xylem (Sun et al. 2022). Despite their capacity to invade the xylem, these viruses often exhibit distinct accumulation patterns and may demonstrate variations in movement direction and speed that are independent of the phloem sap flow. Although viral entry into the xylem could occur through interactions with bacteria or fungi during inoculation, the specific mechanisms facilitating this entry and the ability of these viruses to directly infect plant cells through this process remain poorly understood and require further investigation. Current models suggest that for a virus to access the xylem, it must first infect cells adjacent to immature xylem. As these cells mature, the virus may enter the transpiration stream and exit plant tissues through guttation (Sun et al. 2022). However, the mechanism by which viruses move from the mature xylem into plant living tissues remains poorly understood.

To access distant tissues, viruses must enter the phloem long-distance translocation stream, typically following a stage of cell-to-cell movement within the infected leaf. While it is generally assumed that this entry occurs passively, once the virus reaches the companion cells of minor veins in source leaves, some viral species either fail to enter the phloem or do so with limited efficiency. For instance, some *Brevipalpus* mite-transmitted species of *Dichorhavirus* often remain confined to the inoculated leaves, stems, and fruits, likely due to their inability to access the phloem (Cruz-Jaramillo et al. 2014; Dietzgen et al. 2018). Additionally, plant defense mechanisms can impede viral entry into the phloem, such as that mediated by the lectin jacalin, although the mechanism underlying this phenomenon has not yet been elucidated (Cosson et al. 2010). Furthermore, virus-induced gene silencing may restrict viral access to young and meristematic tissues, a topic explored in more detail below.

Tissue specificity of plant viruses is influenced by host factors. Certain host genotypes have mechanisms that limit the spread of viruses to tissues. For example, in *Manihot esculenta* genotypes resistant to Cassava brown streak virus (*Ipomovirus brunusmanihotis*), the virus is confined to the phloem, preventing its spread to other tissues (Sheat et al. 2021). Similarly, in citrus species resistant to Citrus tristeza virus (CTV, *Closterovirus tristezae*), the virus accumulates in the roots, unlike in susceptible species, where it primarily localizes in the phloem (Harper et al. 2014). Host adaptation also plays a pivotal role in determining viral tropism. For instance, Euphorbia mosaic virus (*Begomovirus euphorbiamusivi*) exhibits phloem-limited tropism in its natural host (*Euphorbia heterophylla*), whereas in an experimental host (*Datura stramonium*), the virus is less restricted, enabling it to infect a broad range of tissues (Rothenstein et al. 2007).

A few soil-borne viruses cause visible symptoms in roots or underground plant organs. For example, Beet necrotic yellow vein virus (*Benyvirus necrobetae*) infects sugar beet and causes rhizomania disease, typically characterized by an increased production of lateral roots and rootlets, resulting in a “bearded” appearance, and severely stunted taproots (Tamada 2016). Similarly, Potato mop-top virus (*Pomovirus solani*) induces brown arcs or rings in the flesh of potato tubers (Falloon et al. 2024).

## Host range vs. host tropism

In plant virology, it is essential to distinguish between host range and host tropism, as they refer to distinct but complementary aspects of virus–host interactions. Host range defines the set of plant species or genotypes in which a virus can infect and complete its replication cycle, either under natural conditions or experimental inoculation (Moury et al. 2017). This property reflects the ability of plant viruses to overcome interspecific barriers, such as compatibility with cellular receptors, suppression of antiviral responses, and successful exploitation of host cellular machinery. In contrast, host tropism describes the differential efficiency with which a virus replicates, accumulates, and systemically spreads among compatible hosts within its host range (McFadden et al. 2009).

For example, Cucumber mosaic virus (CMV, *Cucumovirus CMV*) has a broad host range, infecting over 1,200 species across 521 genera and 100 families, and is transmitted by more than 80 aphid species, by seeds, mechanical inoculation, and parasitic plants such as *Cuscuta* spp. (Jacquemond 2012). However, CMV exhibits differential host tropism, replicating efficiently and causing severe symptoms in some hosts, while remaining asymptomatic or poorly systemic in others. Conversely, viruses with narrow host ranges, such as Beet necrotic yellow vein virus (BNYVV, *Benyvirus necrobetae*) and *Mastrevirus*, typically infect only a few species (e.g., *Chenopodiaceae* or *Poaceae*), but do so consistently and with high efficiency—displaying uniform host tropism (Hugo et al. 1996; Wu et al. 2008). However, these patterns are not universal; viruses with broad host ranges may exhibit uniform host tropism, while those with narrow host ranges can also display differential tropism depending on host compatibility and virus–host interactions.

Most plant viruses have a broad host range. Differences in host-specific permissiveness, antiviral defense pathways, or tissue accessibility contribute to this heterogeneity. Tropism can also vary within the same viral taxon and host plant. For instance, begomoviruses such as Abutilon mosaic virus (AbMV, *Begomovirus bauri*), African cassava mosaic virus (*Begomovirus manihotis*), and TGMV initially exhibit tropism for vascular tissue in *Nicotiana benthamiana*. However, while AbMV remains in the phloem, *B. manihotis* and *B. solanumaureimusivi* spread to other tissues, causing more severe symptoms (Wege et al. 2001). Additionally, variants of plant viruses, including CTV, Broad bean wilt virus 1 (*Fabavirus alphaviciae*), and Alfalfa mosaic virus (*Alfamovirus AMV*), show different symptoms and severity in various hosts due to genetic variations within the viruses and their interactions with the host plants (Carpino et al. 2019; Calderón-Pérez et al. 2019). Understanding host tropism, beyond just host range, is essential for deciphering viral fitness, disease emergence potential, and for designing host-targeted resistance strategies.

## Molecular interactions and host factors shaping viral tropism

Viruses exploit host machinery for replication and movement, with host transcription factors playing important roles in viral DNA replication and inhibition. In particular, ERF, bZIP, WRKY, NAC, MYB, and AP2/ERF families influence viral tropism through their involvement in plant defense mechanisms and gene regulation (Viswanath et al. 2023). Thus, plant viruses can modify the subcellular localization of host factors. Critical host factors involved in plant tropism include actin cytoskeleton components, endosomal sorting complexes, eukaryotic translation factors, glyceraldehyde 3-phosphate dehydrogenase, heat shock proteins, nuclear shuttle protein-interacting kinases, plasmodesmata-located proteins, Rab GTPases, RNA helicases, SNARE proteins and other host factors (Table 1). However, plants have several antiviral defense mechanisms, including innate immunity, translational repression, ubiquitination, autophagy, and ribointerference (Wu et al. 2019; Medina-Puche and Lozano-Duran 2019). Innate immunity, mediated by pathogen-triggered and effector-triggered immunity, induces a hypersensitivity response that limits infection to local tissues and triggers transgenerational systemic resistance mediated by salicylic acid (Ding et al. 2022). Translational repression involves ribosome-inactivating proteins that inhibit protein synthesis in virus-infected cells (Citores et al. 2021). Ubiquitination and autophagy, as two major protein-degradation pathways, act as essential components of the plant antiviral defense system by facilitating the degradation of viral proteins (Sun et al. 2023). These antiviral mechanisms may exhibit specificity against viruses or vary in expression across different plant tissues, thus influencing viral accumulation patterns.

**Table 1:** Host Factors Involved in Plant Virus Tropism

Host factor	Function	Interaction with plant viruses in tropism context	Examples	References
Actin Cytoskeleton Components (ACTIN, Myosin, Formins)	Facilitate intracellular trafficking and movement	Viruses exploit cytoskeleton dynamics for intracellular transport	*Caulimovirus, Tobamovirus*	Van Gisbergen and Bezanilla 2013; Duan and Tominaga 2018
Endosomal Sorting Complexes (ESCRT-I, ESCRT-III)	Mediate membrane trafficking and vesicle formation	Formation of viral replication compartments	*Bromovirus, Tombusvirus*	Barajas et al. 2009; Diaz et al. 2015
Eukaryotic Translation Factors (eIF(iso)4E, eIF4E)	Facilitate translation initiation	Essential for viral RNA recruitment to ribosomes	*Potyvirus*	Zhou et al. 2024
Glyceraldehyde 3-Phosphate Dehydrogenase (GAPDH)	Regulates glycolysis and RNA stability	Involved in maintaining viral RNA ratios and moves to replication sites	*Tombusvirus*	Huang and Nagy 2011
Heat Shock Proteins (HSP70, HSP90)	Chaperones that stabilize viral replication complexes and assist protein folding	Relocate from the cytoplasm to replication sites	*Tombusvirus*	Wu et al. 2023
Nuclear Shuttle Protein-Interacting Kinase (NIK)	Regulates nuclear-cytoplasmic signaling	Redirected by viruses to suppress host immune responses	*Begomovirus*	Martins et al. 2020
Plasmodesmata-located proteins (PDLPs)	Regulate plasmodesmata permeability	Targeted by viral MPs to increase intercellular transport	*Higrevirus,* *Nepovirus,* *Geminivirus*	Amari et al. 2010; Lazareva et al. 2021; Zhao et al. 2023
Rab GTPases	Mediate vesicular transport	Redirected to peroxisomes and mitochondria during viral infection	*Tombusvirus,*	Xu and Nagy 2016
RNA Helicases (AtRH8, AtRH9)	Assist viral RNA unwinding	Move to chloroplasts upon viral infection	*Potyvirus*	Huang et al. 2009; Li et al. 2016
SNARE (VAP27)	Membrane tethering proteins involved in replication complex anchoring	Anchoring replication complexes to ER-derived vesicles and contributing to membrane remodeling	*Potyvirus*	Schaad et al. 1997

RNA interference (RNAi), a conserved eukaryotic defense mechanism, is activated by viral RNA genome and constitutes the main antiviral mechanism in plants. RNA replication intermediates are crucial for the RNAi process. DICER-LIKE (DCL) proteins process double-stranded RNA (dsRNA) into small interfering RNAs (siRNAs) (Ding et al. 2004). These siRNAs are stabilized by the HUA ENHANCER 1 protein, loaded into the RNA-induced silencing complex (RISC), and guide the degradation of complementary viral RNA (Hung and Slotkin 2021). ARGONAUTE (AGO) proteins are crucial for RISC activity, whereas other plant proteins including host RNA-dependent RNA polymerases, amplify siRNA signals (Yang and Li 2018). Additionally, RNAi can induce RNA-directed DNA methylation, suppressing viral transcription (Erdmann and Picard 2020). RNA-binding proteins also contribute to plant defense by directly or indirectly targeting viral RNAs via specialized RNA-binding domains (Musidlak et al. 2017).

Viruses counteract RNAi through the evolution of viral RNA silencing suppressors (VRS) that inhibit RNAi machinery to prevent siRNA production, sequester siRNA duplexes, interfere with AGO and DCL protein functions, and alter epigenetic modifications. VRS also targets additional mechanisms such as trans-acting siRNAs (tasiRNAs) and RNA decay pathways (Jin et al. 2021). Examples include HC-Pro from *Potyvirus* and P19 from *Tombusvirus*. RNAi is active in most plant tissues, with high activity in reproductive, seminal, and meristematic tissues, potentially explaining reduced occurrence of viral tropism in these tissues (Bradamante et al. 2021).

The outcome of viral infection is strongly shaped by the interaction between RNAi and VSRs, affecting viral tropism. Spatial variation in RNAi activity, often higher in meristematic, reproductive, or vascular tissues, can restrict viral accumulation, unless efficiently counteracted by VSRs. Some studies have shown that VSRs not only differ in their silencing suppression potency but also in their tissue-specific effectiveness, influencing whether a virus can establish infection in certain organs (Carluccio et al. 2018; Atabekova et al. 2023). For example, the AC4 protein of Mungbean yellow mosaic virus (*Begomovirus vignaradiatae*) suppresses systemic silencing via siRNA sequestration at the plasma membrane, while the γb protein of Barley stripe mosaic virus (*Hordeivirus hordei*) modulates its suppressor activity through phosphorylation, altering symptom development and systemic spread (Zhang et al. 2018; Carluccio et al. 2018).

## Symptomatology and cellular tropism

Plant viruses induce diverse metabolic and physiological alterations in their hosts, including reduced photosynthesis, elevated respiration rates, accumulation of nitrogen compounds, and increased oxidase activity. Understanding symptom development is challenging due to unidentified intracellular replication sites and the specific metabolic byproducts associated with viral activity (Jiang and Zhou 2023). Viruses exploit host translational machinery for replication and assembly, imposing significant metabolic costs. For example, during TMV infection, virions may constitute over 1% of the fresh weight and up to 50% of the total protein content in infected leaves (Siegel et al. 1978). At the cellular level, viral infection causes subcellular localization changes in host proteins, redirecting them to viral replication sites. RNA viruses replicate in cellular compartments such as the endoplasmic reticulum, chloroplasts, mitochondria, and peroxisome membranes, whereas DNA viruses replicate in the nucleus (Rodriguez-Peña et al. 2021). Interestingly, the severity of disease symptoms frequently does not correlate with viral load (Culver and Padmanabhan 2007). Developing tissues tend to exhibit higher viral load accumulation, though these levels vary significantly across viral species (Cecchini et al. 2002).

Viral infections are associated with transcriptional reprogramming, disruptions in plant hormonal pathways, and the accumulation of metabolites and antioxidant compounds that lead to disease phenotypes (Paudel and Sanfaçon 2018). For instance, chlorosis is related to virus-induced modifications and repression of photosynthetic genes, resulting in changes in the number, size, or structure of chloroplasts (Zhao et al. 2016; Bhattacharyya and Chakraborty 2018). Additionally, epigenetic factors, such as RNA-directed DNA methylation (RdDM), can influence symptom development (Leone et al. 2020).

Symptoms caused by plant viruses include developmental abnormalities (e.g., leaf, shoot, and root deformations, stunting), foliar changes (e.g., chlorosis, mosaic, yellowing, banding, mottling, ring spots, and local or systemic necrosis), and in severe cases, plant death. These symptoms may originate at the infection site or spread through the plant vascular system, and can result either directly from viral infection or as a consequence of the host defense responses (Culver and Padmanabhan 2007; Pallas and García 2011). In contrast, latent viruses do not induce visible symptoms, particularly in wild plants (Takahashi et al. 2019). Many plants harbor endogenous pararetroviruses (DNA retroviruses, *Caulimoviridae* family) that, upon excision from the host genome, can initiate acute infections in new hosts and facilitate horizontal gene transfer. Over evolutionary timescales, this process may increase genetic diversity of their plant hosts (Roossinck 2011). Notably, some plant pararetroviruses persist as endogenous elements that are integrated into the host genome without inducing symptoms. However, abiotic stress or other conditions can activate these integrated viral sequences, leading to episomal virus formation and disease onset (Ishwara Bhat et al. 2023).

## Plant-virus interactions in an ecological context

**Fig. 3:**
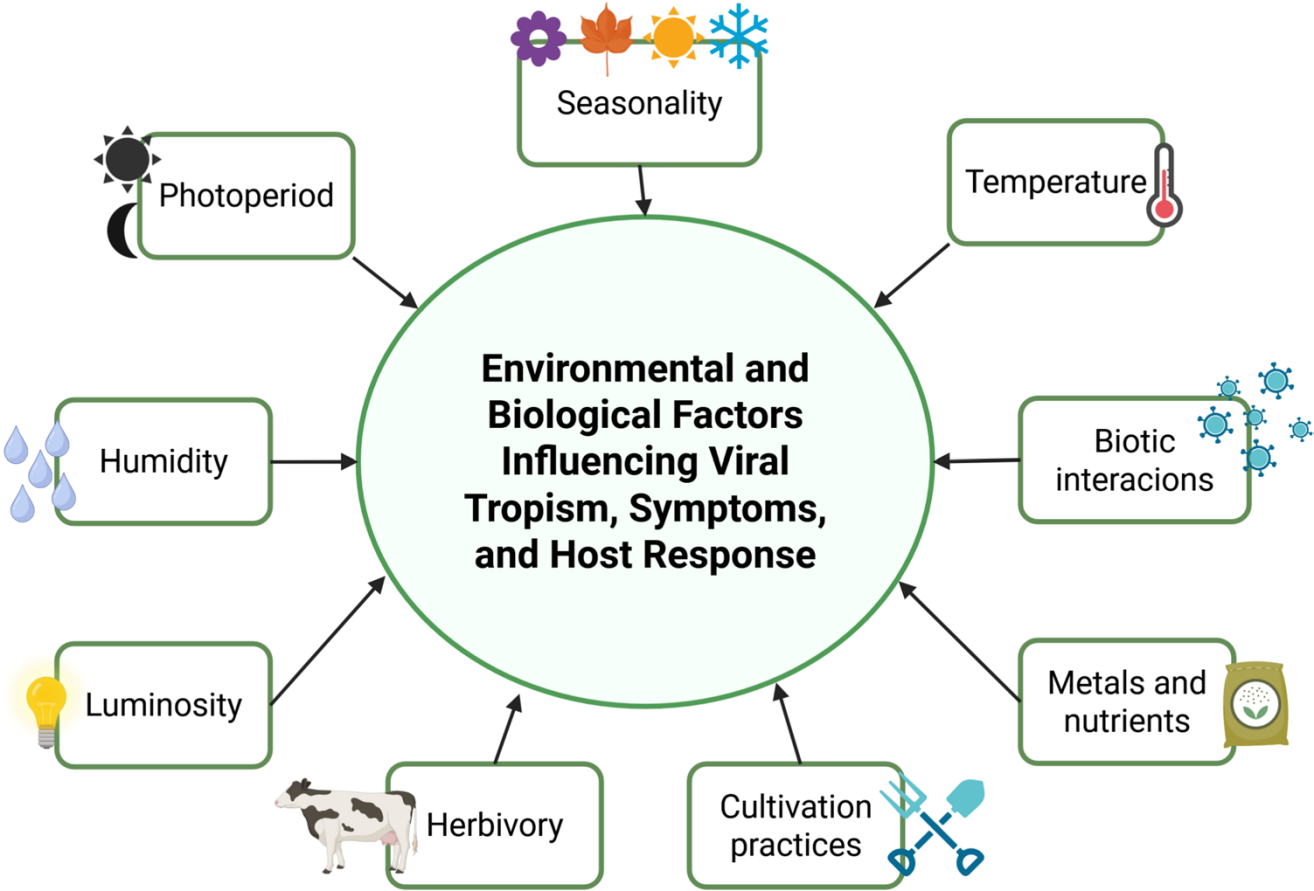
Factors influencing viral symptomatology and tropism. Several environmental and biological factors impact viral symptomatology, host response, and tropism in plants. Key factors include seasonality, temperature, photoperiod, humidity, and luminosity, along with biotic interactions such as co-infection. Additional factors such as herbivory, cultivation practices, metals and nutrients play a crucial role in determining viral infection dynamics and host specificity

Interactions between viruses and their host plants are influenced by environmental and biotic factors, including seasonality, temperature, humidity, photoperiod, light intensity, herbivory, agricultural practices, symptom expression, host response to infection, and viral tropism (Salomon and Seifers 2000; Aranda and Freitas-Astúa 2017) (Fig. 3). For instance, in *Arabidopsis helleri,* TuMV accumulation in young leaves decreases during winter. Meanwhile, antiviral defense mechanisms such as RNAi and induced systemic resistance are activated in autumn and spring, respectively (Honjo et al. 2020).

In the presence of the heavy metal cadmium, at non-toxic levels, systemic infection with Turnip vein clearing virus (*Tobamovirus rapae*) can be blocked by the activation of glycine-rich proteins, highlighting the role of environmental stressors in modulating viral infections (Ueki and Citovsky 2002). Temperature significantly influences plant viral tropism and infection dynamics. At low temperatures, RNAi is inhibited, increasing viral susceptibility and facilitating systemic infections (Szittya 2003). For decades, it has been known that high temperatures can elicit varied effects: some viruses, such as TMV, replicate at 36 °C, albeit with reduced lesion formation, while others, such as CMV, fail to multiply (Kassanis 1952). Temperature also modulates tropism in mixed infections; for example, interactions between Pepino mosaic virus (*Potexvirus pepini*) strains in tomato plants shift from neutral to antagonistic depending on the temperature (Alcaide et al. 2021).

Biotic interactions such as mixed infections can modulate viral tropism, often altering tissue specificity compared with single infections (Mascia and Gallitelli 2016). Representative examples of changes in tropism induced by co-infection are summarized in Supplementary Table 2. Plant virus co-infections can result in synergistic, antagonistic, or neutral interactions which may profoundly affect viral tropism. These outcomes arise through mechanisms such as transcomplementation, RNA silencing, or altered vector interactions, ultimately modulating tissue specificity and host compatibility. Importantly, the impact on tropism is dynamic and context-dependent, influenced by host genotype, virus identity, plant developmental stage, and infection timing (Singhal et al. 2021). Synergistic interactions may facilitate access to new tissues or hosts by suppressing host defenses or by enabling helper-dependent replication (Sánchez-Tovar et al. 2025). In contrast, antagonistic interactions may restrict tropism through competition for cellular machinery, interference between silencing suppressors, or superinfection exclusion, as observed in cross-protection phenomena (Singhal et al. 2021). The sequence of infection is also critical: Moya-Ruiz et al. (2024) demonstrated that slight differences in infection timing between *Polerovirus CABYV* (CABYV) and *Potyvirus citrulli* (WMV) as well as CABYV and *Potyvirus cucurbitaflavitesselati* (ZYMV) in melon altered viral load, tissue colonization, and transmission efficiency.

Vector-transmitted plant viruses exhibit specific tropism in vector tissues, classified based on transmission modes. In non-circulative transmission, the virus remains outside internal cavities and does not cross cell barriers. Viral particles are retained in the vector stylet or anterior gut. If viral retention is brief (seconds to minutes), it is classified as non-persistent. However, if viral retention lasts from minutes to hours, it is semi-persistent. In contrast, circulative or persistent transmission involves virus crossing cell barriers, reaching body cavities, and spreading to the salivary glands. Non-propagative viruses circulate within the vector without replication, whereas propagative viruses multiply inside the vector (Casteel and Falk 2016; Mauck et al. 2018).

Differential viral accumulation patterns in vectors are often specific. For example, Tomato spotted wilt virus (*Orthotospovirus tomatomaculae*) accumulates predominantly in the midgut, foregut, and salivary glands of the thrips *Frankliniella occidentalis* (Nagata et al. 1999). Banana bunchy top virus (*Babuvirus musae*), transmitted by the aphid *Pentalonia nigronervosa*, accumulates in the hemolymph and salivary glands (Watanabe and Bressan 2013). Additionally, some *Rhabdovirus*, *Tenuivirus*, and *Reovirus* can infect vector reproductive structures and exhibit transovarial transmission (Huo et al. 2014).

Plant viral tropism in insects is determined by the presence of viral receptors and specific tissue-related proteins in vector cells, which vary depending on taxonomic group and the viral transmission mode. For example, in *Bemisia tabacci* (whitefly), proteins such as myosins, peptide receptors, heat shock proteins, knottins, sugar, amino acids, and ATP-binding cassette transporters are differentially expressed in the presence of various plant viruses (Catto et al. 2022).

## Plant viral transmission mechanisms

Plant viruses use two primary transmission mechanisms: horizontal and vertical. Horizontal transmission is directly linked to viral tropism, as viral particles must colonize various tissues, depending on their point of delivery. In contrast, vertical transmission presents an additional challenge, requiring viruses to mobilize and exhibit specific tropism toward reproductive and seed tissues to successfully colonize these structures and ensure their dissemination to the next generation. In this section, we explore these mechanisms in detail.

### Horizontal transmission

**Fig. 4:**
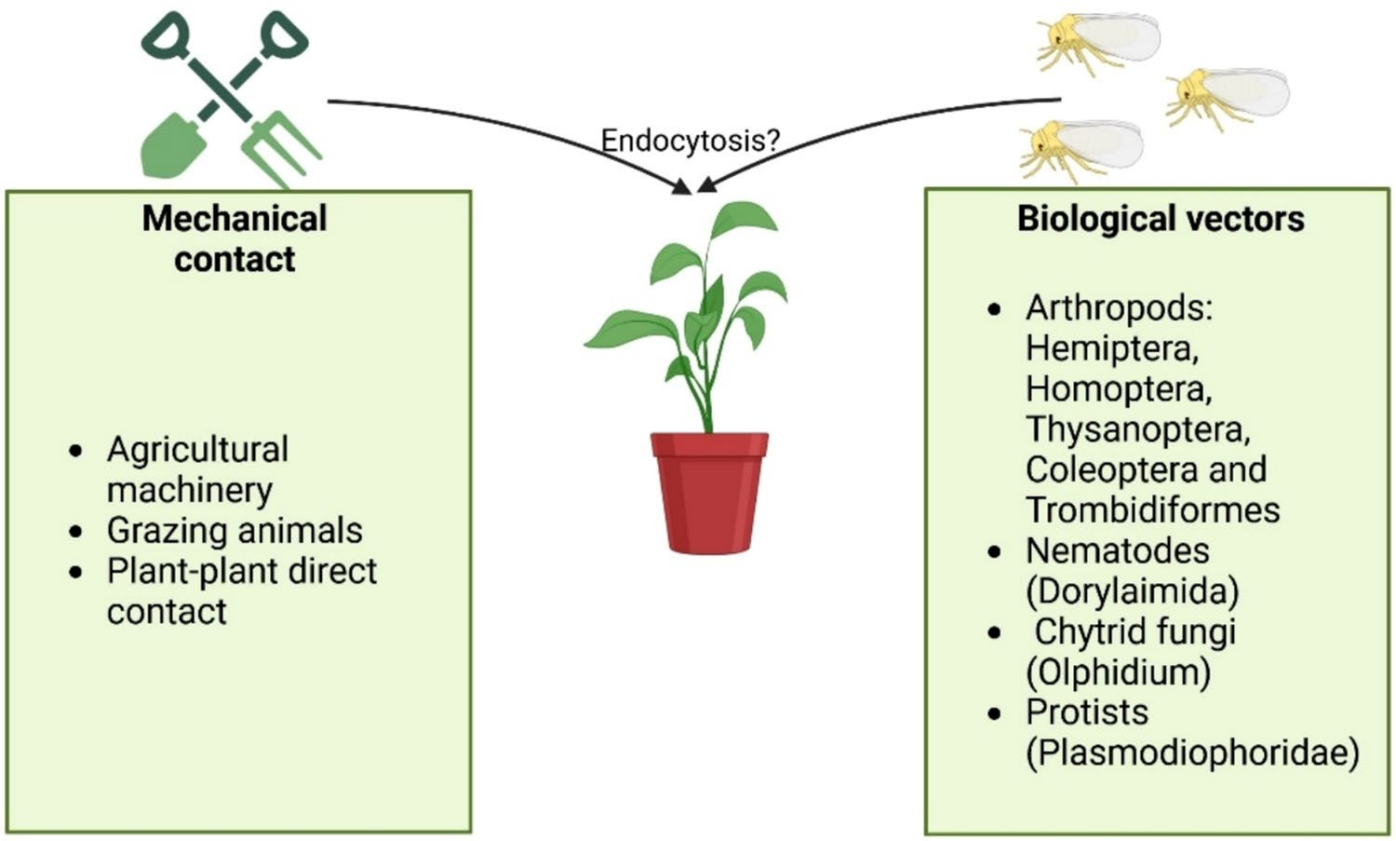
Mechanisms of horizontal transmission of plant viruses. Horizontal transmission in plant viruses occurs through mechanical contact or biological vectors. Mechanical transmission includes agricultural machinery, grazing animals, and direct contact between plants. Biological vectors encompass arthropods (e.g., Hemiptera, Homoptera, Thysanoptera, Coleoptera, Trombidiformes), nematodes (Dorylaimida), chytrid fungi (*Olphidium* sp.), and protists (Plasmodiophoridae)

Plant viruses are transmitted horizontally through mechanical contact (e.g., agricultural machinery, grazing animals, and direct plant-plant contact) or biological vectors (Roossinck 2013). Biological vectors include arthropods such as aphids, whiteflies, thrips, beetles, and mites (Ng and Falk 2006; Brault et al. 2010). Among these, aphids are the most prevalent vectors, responsible for transmitting over 55% of plant viruses, with more than 200 aphid species identified as viral vectors. Other biological vectors include nematodes, chytrid fungi, and protists (Astier et al. 2001; Singh et al. 2008, 2020) (Fig. 4).

Horizontal transmission mechanisms play a crucial role in viral tropism, as different transmission modes are associated with specific target tissues where viruses are delivered. Viruses transmitted via mechanical contact or by chewing insects generally localize to epidermal and mesophyll parenchyma cells, while those transmitted by piercing-sucking insects are directly delivered into the vascular tissue (Ueki and Citovsky 2006). In contrast, viruses transmitted by nematodes, chytrids, or protists initially encounter root cells upon infection (Andika et al. 2016). This establishes a key link between the type of horizontal transmission and viral tropism, as viruses must adapt and develop effective strategies to colonize the different tissues that they encounter during the infection process.

### Vertical transmission

Vertical transmission occurs when an infected parent plant transmits the virus to its offspring, typically via gametes (pollen or ovules) or embryos, as well as through the infection of seedlings after germination (Bradamante et al. 2021). Infection rates in vertical transmission vary widely, ranging from 0.2% to 99%. This mode of transmission is associated with cryptic viruses, such as those in the *Partitiviridae* family or the *Endornavirus* genus. These viruses are asymptomatic, lack cell-to-cell movement, and accumulate in meristems and reproductive tissues, with the host RNAi machinery maintaining the infection. Interestingly, cryptic viruses have been shown to enhance crop yield and productivity under certain conditions (Roossinck 2010; Fukuhara 2019). Comparative studies in Arabidopsis have revealed generational changes in vertically transmitted CMV, reducing viral accumulation and virulence, unlike horizontal transmission (Pagán et al. 2014).

Plant viruses are commonly unable to colonize meristems and seeds (Moreno and López-Moya 2020), suggesting regulatory systems that control RNA entry into meristems to protect them against viral invasion (Foster et al. 2002). Virus-free regions within meristems, ranging in size from 100 to 1000 µm, can be exploited to produce virus-free plants through in vitro culture or grafting techniques (Agüero et al. 2013).

However, recent findings suggest that viral exclusion from meristems depends on multiple factors, including host antiviral responses and virus-specific strategies. Some viruses, such as TRV, have been shown to invade and replicate within meristematic tissues (Valentine et al. 2004). In contrast, other viruses may persist in meristematic cells at undetectable levels due to limited replication or suppression by RNA interference (RNAi) mechanisms (Bradamante et al. 2021). This suggests that viral tropism in meristems is not uniform and may vary depending on the virus-host interaction and mode of transmission. Virus accumulation patterns within meristems can vary, including complete exclusion from meristematic stem cells, restriction to the meristem vascular system, full invasion of the meristem including meristematic stem cells, or localization in outer meristematic layers (L1 and L2) and primordia of leaves or flowers (Ishwara Bhat et al. 2023). The ability of certain viruses to persist in meristems without active replication could represent a form of latent tropism, where viruses evade immune surveillance and elimination during vegetative propagation but remain capable of re-emerging under favorable conditions.

## Plant viruses targeting seeds as a case of specialized tropism

The presence of plant viruses in seeds can impact seedling development, leading to cellular ultrastructural malformations and impaired functions of structures such as chloroplasts (Harsányi et al. 2005). The presence of plant viruses in seeds has been reported in approximately 25% of known plant viruses, although their localization within seed structures varies (Escalante et al. 2024). The suspensor— a long cell chain that supports the developing embryo — is the primary route for viral entry during a specific developmental window (Navarro et al. 2019). For instance, in maize infected with Sugarcane mosaic virus (*Potyvirus sacchari*), embryo susceptibility decreases during late-stage development due to apoptosis of the suspensor cells (Li et al. 2007). However, not all seed-transmitted viruses infect the embryo directly. *Tobamovirus*, mainly located in the seed coat, lead to seedling infection only after germination. The presence of viruses in reproductive organs can also affect seed and pollen development, with virions accumulating in pollen grains, stamens, and ovaries (Ishibashi et al. 2023). Low-virulence viruses that are vertically transmitted, such as Pea seed-borne mosaic virus (*Potyvirus pisumsemenportati*), may bypass host defenses more effectively. These viruses experience genetic drift and narrower bottlenecks during seed transmission, a process that can exceed the genetic variation observed in horizontally transmitted viruses (Fabre et al. 2014). Several factors affect viral seed transmission, including cultivar type, maternal plant age, and environmental conditions, alongside embryo or gamete infection pre-fertilization (Pagán 2022).

Viral persistence and transmission through seeds require circumvention of host immune mechanisms, particularly RNAi, which is highly active during reproductive development. Recent evidence demonstrates that RNAi can block the vertical transmission of viruses, such as CMV, and that viruses must deploy suppressors of RNAi to overcome this barrier (Liu and Ding 2024). These immune evasion strategies are shaped by evolutionary trade-offs between horizontal and vertical transmission routes, which influence viral fitness and tissue tropism in the reproductive organs (García-Ordóñez and Pagán 2024). In addition, genes such as *DCL* and *RDR6* have been identified to contribute to antiviral defenses in Arabidopsis and soybean seeds. Genome-wide association studies have highlighted several genes including *HSP20-like*, *ZAT8*, *LURP1*, *GMD1*, *PLL18*, *P4H11*, *RTFL13*, *ORTHL*, *CIPK2* and *MAC5C,* linked to stress response and cell wall metabolism, and may influence viral transmission (Escalante et al. 2024). Additionally, the *L3* gene has been shown to modify seed transmission resistance of Tomato brown rugose fruit virus (ToBRFV, *Tobamovirus fructirugosum*), yielding mixed results across pepper species (Matsushita et al. 2024). In viroids, specific RNA domains facilitate transmission by targeting sporogenous tissues, which feed pollen cells via plasmodesmata, suggesting a pre-meiotic viral entry (Hammond 2017).

Seed transmission can be affected by factors such as genotype, maternal plant age, and environmental conditions, primarily occurring through pre-fertilization gamete infection or post-fertilization embryo invasion (Cobos et al. 2019; Baldodiya et al. 2020). True seed-transmitted viruses are localized within embryonic tissues, whereas seed-borne viruses remain in the seed coat or endosperm (Pagán 2022). There are two routes of embryo infection by seed-transmitted viruses: infection via gametes involved in fertilization and post-fertilization infection through movement in maternal tissues. However, studies on the mechanisms of virus entry and colonization in male and female gametes are scarce. The characterization of meristematic and transgenerational antiviral barriers remains under investigation, with RNAi pathways and stress-related genes playing essential roles in resistance to plant viruses (Domier et al. 2011). Host factors involved in virus transmission and accumulation include uncharacterized loci and genes related to stress responses, embryogenesis, and cell wall metabolism (Cobos et al. 2019; Pagán 2022). Viral determinants to vertical transmission include proteins associated with viral replication, movement, and VRS (Cobos et al. 2019).

In addition, vertically transmitted viruses significantly affect agricultural economies. For example, Arabidopsis accessions globally host Arabidopsis latent virus-1 (*Comovirus arabidopsis*) (Verhoeven et al. 2023). On the other hand, ToBRFV poses a severe threat to tomato crops in over 35 countries due its high prevalence in seed coats (Salem et al. 2016; Zhang et al. 2022). This underscores the urgent need for improved diagnostic, treatment, and resistance strategies, as current methods offer limited durability and efficacy (Pagán 2022).

## Advanced methodologies for investigating plant viral tropism

Recent advancements in microscopy, sequencing, and analytical techniques have significantly expanded the toolkit for studying viral tropism in plants. Single-molecule fluorescence in situ hybridization (smFISH) enables precise localization of viral genomes within specific tissues by using fluorescently labeled probes, allowing single-virus detection at subcellular resolution (Duncan et al. 2017). This technique is particularly valuable for identifying preferential viral accumulation sites and understanding intracellular viral trafficking.

Spatial transcriptomics, which integrates high-throughput RNA sequencing with spatial context, enables transcriptome-wide profiling of virus-host interactions without disrupting tissue organization, providing insights into localized viral replication and host responses (Robles-Remacho et al. 2023). Long-read sequencing technologies, such as Oxford Nanopore and PacBio SMRT sequencing, enable full-length viral genome assembly and the detection of viral quasispecies, recombination events, and epitranscriptomic modifications, providing deeper insights into viral population diversity and tissue-specific variations (Boldogkői et al. 2019). Dual RNA-seq, which simultaneously profiles viral and host transcriptomes, facilitates the identification of tissue-specific gene expression changes upon infection (Westermann et al. 2017). Small RNA sequencing (sRNA-seq) further contributes to understanding plant antiviral RNA interference (RNAi) responses, which shape viral tropism (Golyaev et al. 2019).

Metagenomic and metatranscriptomic sequencing play a crucial role in uncovering the tissue-specific distribution and diversity of viral populations within plants (Rahimian and Panahi 2024). These approaches enable the unbiased detection of viral genomes and transcripts across different organs, allowing for a comparative analysis of viral communities in distinct tissues, such as leaves, roots, flowers, and seeds. By identifying viruses or viral variants that preferentially accumulate in specific plant structures, metagenomic and metatranscriptomic data can reveal key factors influencing viral tissue specificity, systemic movement, and persistence in reproductive organs. Additionally, these approaches facilitate the detection of co-infections, helping us to understand how viral interactions within a plant influence competition, synergy, or exclusion in different tissues. Integrating high-throughput sequencing methodologies with spatial and single-cell approaches provides an unprecedented resolution for mapping plant viral distribution and understanding tissue-specific infection dynamics.

Super-resolution microscopy has emerged as a powerful tool for studying viral tropism in plants, providing nanoscale insights into viral replication complexes, movement proteins, and intracellular transport. These advanced imaging methods surpass the diffraction limit of conventional light microscopy, enabling unprecedented visualization of virus-host interactions. Among these, single-molecule localization microscopy techniques, such as Stochastic Optical Reconstruction Microscopy and Photoactivated Localization Microscopy, achieve resolutions of 20–30 nm, allowing detailed analysis of viral replication sites and movement within plant cells (Schermelleh et al. 2019; Eilts et al. 2023). Expansion microscopy (ExM) further enhances structural visualization by physically expanding biological samples, facilitating imaging of nanoscale viral structures with conventional microscopes. For instance, 12 × 3D-ExM has demonstrated the capability to resolve nuclear pore complexes and virus particles below 30 nm (Norman et al. 2025), which is particularly relevant for studying viral entry and intracellular trafficking. Recent advancements in Airyscan and structured illumination microscopy (SIM) have improved multicolor imaging, allowing 4-color 3D imaging at nanometer resolution, which could be instrumental in elucidating the spatial organization of viral proteins during infection (Eilts et al. 2023). Additionally, deep learning-based single-frame super-resolution microscopy (SFSRM) offers 30 nm spatial resolution with 10 ms temporal resolution, providing real-time tracking of viral movement and host responses (Chen et al. 2023). Complementary to these optical techniques, cryo-electron microscopy (Cryo-EM) and cryo-electron tomography (Cryo-ET) provide near-atomic resolution imaging of virions, replication factories, and virus-induced host organelle modifications, further advancing our understanding of viral pathogenesis *in planta* (Chang et al. 2012; Stass et al. 2018). As these imaging technologies continue to evolve, their integration with computational modeling and live-cell imaging will offer new perspectives on the mechanisms underlying plant viral tropism, host defense strategies, and systemic infection processes.

Nanoscale secondary ion mass spectrometry (NanoSIMS) is a powerful technique for tracing isotopic-labeled viral components and metabolic changes in infected tissues, providing quantitative data on nutrient redistribution and metabolic shifts induced by viral infections (Pett-Ridge and Weber 2022). Multiplexed immunohistochemistry and proximity ligation assays (PLA) allow for in situ detection of viral proteins and their interactions with host factors enabling the mapping of host pathways involved in viral transport and replication (Leuchowius et al. 2011; Manesse et al. 2020).

Regarding non-destructive imaging, such as optical coherence tomography, it offers deep-tissue visualization of systemic viral spread in intact plant organs preserving spatial relationships, critical for understanding phloem-mediated movement and seed transmission (Verboven et al. 2013). Additionally, the integration of these cutting-edge methodologies with machine learning-driven image analysis, artificial intelligence (AI) and computational modeling enhances data interpretation, allowing for predictive modeling of viral dissemination patterns (Wu et al. 2024; Jafar et al. 2024). Particularly, AI tools are being increasingly applied in plant virology to predict structural and functional properties of viral proteins. Notably, AlphaFold2 has enabled high-accuracy 3D modeling of movement proteins, silencing suppressors, and other determinants of host specificity and tissue tropism. In fact, our group previously employed AlphaFold2 to model the structure of the Tm-2^2^ resistance protein and the ToBRFV movement protein, enabling in silico docking and mutagenesis of key residues involved in viral evasion (Rivera-Márquez et al. 2022). Similarly, AlphaFold2 has been applied to predict the structure and multifunctionality of Grapevine fanleaf virus (*Nepovirus foliumflabelli*) proteins, including suppressors and replication-related proteins (Roy et al. 2024), and to study protein–protein interactions between Chilli leaf curl virus (*Begomovirus chillicapsici*) proteins and host proteins in *Capsicum annuum* (Pandey et al. 2024). These approaches are advancing our ability to resolve viral determinants of tropism and their host interactions at high resolution, with significant implications for understanding systemic infections and improving crop protection strategies.

## Conclusions and perspectives

Understanding viral tropism in plants, particularly in reproductive tissues and seeds, remains a major challenge in plant virology. Although recent advances have improved our ability to detect and localize viruses at high resolution, key gaps remain in identifying the molecular determinants that govern tissue-specific accumulation and systemic movement. Vertical transmission through seeds is an efficient viral strategy for persistence and dissemination, but its study is hindered by low viral titers and the need for long-term assays. The advent of high-throughput sequencing and advanced imaging techniques—such as single-cell RNA sequencing and cryo-electron tomography—has expanded our capacity to explore these processes, despite limitations imposed by plant cellular complexity.

Integrating multi-omics data with gene editing tools offers promising avenues for identifying host and viral factors involved in tissue tropism. Future research should aim to elucidate the mechanisms underlying viral movement within reproductive organs and uncover how viruses exploit or bypass tissue-specific barriers. These insights have important translational applications in agriculture. Tropism data can inform the design of resistance strategies based on tissue-specific gene expression, guide breeding programs by identifying vulnerable tissues, and support the selection of grafting combinations that restrict systemic spread. For viruses with strong seed tropism, targeting vertical transmission routes becomes a strategic priority. Moreover, identifying and characterizing host factors involved in viral tropism opens the door to targeted genome editing to disrupt key virus–host interactions. Additionally, plant viruses with defined tropism profiles could be repurposed as vectors for gene delivery or as biocontrol agents.

Looking ahead, combining knowledge of viral tropism with spatial omics and artificial intelligence tools—such as structure prediction and host–pathogen interaction modeling—could uncover novel molecular determinants of tissue-specific infection. Furthermore, characterizing how viral tropism evolves in response to climate change, environmental stressors, and mixed infections will be key to predicting disease emergence in crops. Leveraging these insights will not only enhance crop resilience but also enable the synthetic redesign of viral vectors for precision agriculture and next-generation biotechnological applications.

## Supplementary Information

Below is the link to the electronic supplementary material.

Supplementary file1 (DOCX 40 KB)

## Data Availability

No datasets were generated or analysed during the current study.
