# Supplementary material for: Viral tropism in plants, reproductive tissues, and seeds
Source: Arch Microbiol. 2025 May 23;207(7):152. doi: 10.1007/s00203-025-04353-9 (PMC12098505; doi:10.1007/s00203-025-04353-9)
Supplement: Supplementary file 1 — Supplementary file1 (DOCX 40 KB) [file 203_2025_4353_MOESM1_ESM.docx]

**Supplementary material**

**Supplementary tables**

**Supplementary Table 1.** **Examples of viruses with tissue-specific tropism (phloem-limited)**

| **Common Name** | **Specie** | **Host range** | **Primary transmission vector** | **Family** | **References** |
| --- | --- | --- | --- | --- | --- |
| Beet yellow virus | *Closterovirus flavibetae* | Moderate to broad | Aphids (*Myzus persicae*) | Closteroviridae | Jiménez et al. 2021 |
| Citrus tristeza virus | *Closterovirus tristezae* | Narrow | Aphids (*Toxoptera citricida*) |  | Dawson et al. 2013 |
| Abutilon mosaic virus | *Begomovirus bauri* | Modarately broad | Whiteflies (*Bemisia tabaci*) | Geminiviridae | Rothenstein et al. 2007 |
| Bean golden mosaic virus | *Begomovirus costai* | Narrow |  |  |  |
| Euphorbia mosaic virus | *Begomovirus euphorbiamusivi* | Moderate to broad |  |  |  |
| Indian Cassava mosaic virus | *Begomovirus manihotisindianense* | Moderate to broad |  |  |  |
| Sida micrantha Mosaic virus | *Begomovirus sidamicranthae* | Narrow |  |  |  |
| Squash leaf curl virus | *Begomovirus cucurbitapeponis* | Moderate |  |  |  |
| Tomato yellow leaf curl *virus* | *Begomovirus coheni* | Broad |  |  |  |
| Cucurbit aphid-borne yellows virus | *Polerovirus CABYV* | Moderately broad | Aphids (*Aphis gossypii* and *Myzus persicae*) | Solemoviridae | Schoeny et al. 2020 |
| Potato leafroll virus | *Polerovirus PLRV* | Narrow | Aphids (*Myzus persicae*) |  | Bendix and Lewis 2018 |
| Tobacco necrotic dwarf virus | *Polerovirus TNDV* | Narrow |  |  | Takanami and Kubo 1979 |
| Southern rice black-streaked dwarf virus | *Fijivirus boryzae* | Moderate | Planthoppers (*Sogatella furcifera*) | Spinareoviridae | Zhou et al. 2013 |
| Rice black streaked dwarf virus | *Fijivirus alporyzae* | Narrow | Planthoppers (*Laodelphax striatellus*) |  | Lv et al. 2017 |
| Rice gall dwarf virus | *Phytoreovirus betaoryzae* | Narrow | Planthoppers (*Recilia dorsalis*) | Sedoreoviridae |  |
| Rice ragged stunt virus | *Oryzavirus oryzae* | Narrow | Planthoppers (*Nilaparvata lugens*) | Spinareoviridae |  |

Bendix C, Lewis JD (2018) The enemy within: phloem‐limited pathogens. Mol Plant Pathol 19:238–254. https://doi.org/10.1111/mpp.12526

Dawson WO, Garnsey SM, Tatineni S, et al (2013) Citrus tristeza virus-host interactions. Front Microbiol 4:. https://doi.org/10.3389/fmicb.2013.00088

Jiménez J, Moreno A, Fereres A (2021) Semipersistently Transmitted, Phloem Limited Plant Viruses Are Inoculated during the First Subphase of Intracellular Stylet Penetrations in Phloem Cells. Viruses 13:137. https://doi.org/10.3390/v13010137

Lv M-F, Xie L, Song X-J, et al (2017) Phloem-limited reoviruses universally induce sieve element hyperplasia and more flexible gateways, providing more channels for their movement in plants. Sci Rep 7:16467. https://doi.org/10.1038/s41598-017-15686-x

Rothenstein D, Krenz B, Selchow O, Jeske H (2007) Tissue and cell tropism of Indian cassava mosaic virus (ICMV) and its AV2 (precoat) gene product. Virology 359:137–145. https://doi.org/10.1016/j.virol.2006.09.014

Schoeny A, Rimbaud L, Gognalons P, et al (2020) Can Winged Aphid Abundance Be a Predictor of Cucurbit Aphid-Borne Yellows Virus Epidemics in Melon Crop? Viruses 12:911. https://doi.org/10.3390/v12090911

Takanami Y, Kubo S (1979) Enzyme-assisted Purification of two Phloem-limited Plant Viruses: Tobacco Necrotic Dwarf and Potato Leafroll. J Gen Virol 44:153–159. https://doi.org/10.1099/0022-1317-44-1-153

Zhou G, Xu D, Xu D, Zhang M (2013) Southern rice black-streaked dwarf virus: a white-backed planthopper-transmitted fijivirus threatening rice production in Asia. Front Microbiol 4:. https://doi.org/10.3389/fmicb.2013.00270

**Supplementary Table 2. Examples of viral co-infections associated with altered tissue tropism**

| **Co-infection (Viruses)** | **Interaction Outcome** | **Observed Effect on Tropism** | **References** |
| --- | --- | --- | --- |
| *Begomovirus bauri* (AbMV) + *Cucumovirus CMV* (CMV) | Synergism | CMV enables AbMV to escape phloem restriction and invade mesophyll (palisade and spongy parenchyma) tissues. | Wege and Siegmund 2007 |
| *Begomovirus costai* (BGMV) + *Begomovirus solanumaureimusivi* (TGMV) | Synergism (tissue-specific transcomplementation) | TGMV enables BGMV to escape phloem limitation and invade mesophyll tissue in *Nicotiana benthamiana* | Morra and Petty 2000 |
| *Begomovirus solanumflavusmaculae* (ToYSV) + *Begomovirus solanumrugosi* (ToRM) | Antagonist at initial stage of infection, neutral at systemic infection | ToYSV releases ToRMV from the phloem | Alves-Júnior et al. 2009 |
| *Potyvirus atuberosi* (PVA)+ *Polerovirus PLRV* (PLRV) | Synergism | \|  \| \| --- \|   PLRV titers increase (up to 6×); phloem limitation is alleviated only during coinfection, not by PVA^HC-Pro^ alone. | Savenkov and Valkonen 2001 |
| *Polerovirus BCHV* (BChV*)+ Closterovirus flavibetae* (BYV) | Antagonism (reduced BChV transmission) | BYV alters intracellular distribution of BChV | Khechmar et al. 2024 |
| *Potyvirus cucurbitaflavitesselati* (ZYMV) + *Cucumovirus CMV* (CMV, non-systemic strain) | Synergism | CMV gains long-distance/systemic movement | Choi et al. 2002 |
| *Umbravirus pisi* (PEMV-2) + *Polerovirus PLRV* (PLRV) | Synergism (complementation) | PLRV escapes phloem restriction and invades mesophyll tissue; mechanical transmission becomes possible. | Ryabov et al. 2001 |

AbMV: Abutilon mosaic virus; BChV: Beet chlorosis virus; BGMV: Bean golden mosaic virus; BYV: Beet yellows virus; CMV: Cucumber mosaic virus; PEMV-2: Pea enation mosaic virus 2; PLRV: Potato leafroll virus; PVA: Potato virus A; TGMV: Tomato golden mosaic virus; ToYSV: Tomato yellow spot virus; TRMoV: Tomato rugose mosaic virus; ZYMV: Zucchini yellow mosaic virus

Alves-Júnior M, Alfenas-Zerbini P, Andrade EC, et al (2009) Synergism and negative interference during co-infection of tomato and Nicotiana benthamiana with two bipartite begomoviruses. Virology 387:257–266. https://doi.org/10.1016/j.virol.2009.01.046

Choi SK, Yoon JY, Ryu KH, et al (2002) Systemic movement of a movement-deficient strain of Cucumber mosaic virus in zucchini squash is facilitated by a cucurbit-infecting potyvirus. J Gen Virol 83:3173–3178. https://doi.org/10.1099/0022-1317-83-12-3173

Khechmar S, Chesnais Q, Villeroy C, et al (2024) Interplay between a polerovirus and a closterovirus decreases aphid transmission of the polerovirus. Microbiol Spectr 12:e01115-24. https://doi.org/10.1128/spectrum.01115-24

Morra MR, Petty ITD (2000) Tissue Specificity of Geminivirus Infection Is Genetically Determined. Plant Cell 12:2259–2270. https://doi.org/10.1105/tpc.12.11.2259

Ryabov EV, Fraser G, Mayo MA, et al (2001) Umbravirus Gene Expression Helps Potato leafroll virus to Invade Mesophyll Tissues and to be Transmitted Mechanically between Plants. Virology 286:363–372. https://doi.org/10.1006/viro.2001.0982

Savenkov EI, Valkonen JPT (2001) Potyviral Helper-Component Proteinase Expressed in Transgenic Plants Enhances Titers of Potato Leaf Roll Virus but Does Not Alleviate Its Phloem Limitation. Virology 283:285–293. https://doi.org/10.1006/viro.2000.0838

Wege C, Siegmund D (2007) Synergism of a DNA and an RNA virus: Enhanced tissue infiltration of the begomovirus Abutilon mosaic virus (AbMV) mediated by Cucumber mosaic virus (CMV). Virology 357:10–28. https://doi.org/10.1016/j.virol.2006.07.043
